# Liver and atherosclerotic risks associated with cryptogenic steatotic liver disease: are the new diagnostic criteria justified?

**DOI:** 10.1097/MS9.0000000000005208

**Published:** 2026-06-02

**Authors:** Dua Rasool, Sameer Ahmad, Aayush Visaria, Ghulam Husain Abbas

**Affiliations:** a Department of Medicine, Foundation University Medical College, Islamabad, Pakistan; b Department of Medicine, Rutgers, Robert Wood Johnson Medical School, New Brunswick, New Jersey, USA; c Faculty of Medicine, Ala-Too International University, Bishkek, Kyrgyz Republic

*To the Editor,*

In June 2023, a new nomenclature for steatotic liver disease (SLD) was introduced, replacing the term non-alcoholic fatty liver disease (NAFLD) with metabolic dysfunction-associated steatotic liver disease (MASLD) under the broader SLD umbrella. Within this classification, MASLD is distinct from cryptogenic SLD and other subtypes. The article by Wang *et al*[1] demonstrated that cryptogenic SLD is associated with lower liver and atherosclerotic risks compared with MASLD. We commend the authors for their contribution to validating the new SLD classification. Nevertheless, the strategies used to assess alcohol consumption, control potential confounders, and evaluate liver outcomes may have limitations, which warrant further consideration in future research.

First, the study excluded frequent drinkers while retaining non-drinkers, social drinkers, and former drinkers when comparing MASLD and cryptogenic SLD. Restricting the cohort to confirmed non-drinkers would better eliminate alcohol effects. Furthermore, although the Taiwan Biobank is a valuable resource, it relies on self-reported alcohol use and limited clinical detail, which restricts its ability to disentangle alcohol-related injury from other metabolic or idiopathic causes of liver disease[2]. Determining alcohol measurement and highly sensitive and specific alcohol biomarkers, such as phosphatidylethanol and sialylation of Apo J, to evaluate both acute and chronic alcohol intake, along with more detailed clinical assessments in future studies, may help overcome this limitation[2]. It has been shown that some patients with NAFLD may present with metabolic parameters within normal or lower than conventional cut-off values[3], and individuals with cryptogenic cirrhosis demonstrate a higher prevalence of NAFLD risk factors compared to those with viral hepatitis-related cirrhosis[4]. Combined with evidence that no level of alcohol consumption is entirely safe, it remains uncertain whether cryptogenic SLD represents a truly idiopathic condition or instead reflects liver disease influenced by subtle metabolic abnormalities below diagnostic thresholds or unrecognized alcohol exposure[5]. Future studies are needed to better define the risk factors associated with cryptogenic SLD.

HIGHLIGHTS

- The steatotic liver disease (SLD) classification (2023) distinguishes metabolic dysfunction-associated SLD from cryptogenic SLD, with evidence suggesting lower liver and atherosclerotic risks in cryptogenic SLD.
- Alcohol consumption assessment is a major limitation, as reliance on self-reported data and the inclusion of social or former drinkers may bias the results.
- Cryptogenic SLD may not be truly idiopathic, potentially reflecting subclinical metabolic dysfunction or unrecognized alcohol exposure.
- Confounding factors were insufficiently controlled, with missing variables such as comorbidities, lifestyle, and family history.
- Use of fatty liver index and non-alcoholic fatty liver disease fibrosis score scores may be problematic as they include metabolic parameters already used in disease classification, reducing diagnostic clarity.
- Future research should improve alcohol measurement, confounder control, and liver assessment tools (e.g., elastography) to strengthen validity.

Second, although the authors used propensity score matching for age and/or gender, additional variables such as comorbidities, family history of liver steatosis or obesity, and socio-behavioral factors (diet, exercise, smoking, and medication adherence) warrant inclusion to further minimize confounding^[^6,7^]^.

Third, hepatic biomarkers and scoring systems, such as the fatty liver index and the NAFLD fibrosis score (NFS), were used to evaluate liver outcomes. However, because these scores incorporate metabolic parameters, such as body mass index, which are also used to distinguish MASLD from cryptogenic SLD, their application in this context may be limited. Furthermore, the NFS includes an indeterminate category in which patients cannot be reliably classified into low- or high-probability groups for significant fibrosis. Ultrasound-based elastography represents a feasible, non-invasive alternative for assessing liver fibrosis and may provide greater diagnostic precision[8].

Overall, the authors’ thorough investigation of hepatic and atherosclerotic risks in MASLD and cryptogenic SLD patients is noteworthy. Future studies should place greater emphasis on the assessment of alcohol consumption, the control of broader confounders, and the use of more reliable measures of liver outcomes to enhance the robustness and credibility of future findings.

## Data Availability

Not applicable.
